# Towards an Advanced Modeling of Hybrid Composite Cutting: Heat Discontinuity at Interface Region

**DOI:** 10.3390/polym15081955

**Published:** 2023-04-20

**Authors:** Brahim Salem, Ali Mkaddem, Sami Ghazali, Malek Habak, Bassem F. Felemban, Abdessalem Jarraya

**Affiliations:** 1 LA2MP, National School of Engineering of Sfax, University of Sfax, Sfax 3038, Tunisia; 2 Department of Mechanical and Materials Engineering, FOE, University of Jeddah, Jeddah 21589, Saudi Arabia; 3 LTI, Avenue des Facultés Le Bailly, Université de Picardie Jules Verne, CEDEX 1, 80001 Amiens, France; 4 Department of Mechanical Engineering, College of Engineering, Taif University, Taif 21955, Saudi Arabia

**Keywords:** cutting, CFRP/Ti, temperature, interface, damage, VUMAT, VDFLUX

## Abstract

In this study, a thermomechanical model is developed to simulate a finite drilling set of Carbon Fibre Reinforced Polymers (CFRP)/Titanium (Ti) hybrid structures widely known for their energy saving performance. The model applies different heat fluxes at the trim plane of the two phases of the composite, owing to cutting forces, in order to simulate the temperature evolution at the workpiece during the cutting step. A user-defined subroutine VDFLUX was implemented to address the temperature-coupled displacement approach. A user-material subroutine VUMAT was developed to describe Hashin damage-coupled elasticity model for the CFRP phase while Johnson–Cook damage criteria was considered for describing the behavior of titanium phase. The two subroutines coordinate to evaluate sensitively the heat effects at the CFRP/Ti interface and within the subsurface of the structure at each increment. The proposed model has been first calibrated based on tensile standard tests. The material removal process was then investigated versus cutting conditions. Predictions show discontinuity in temperature field at interface that should further favor damage to localize especially at CFRP phase. The obtained results highlight the significant effects of fibre orientation in dominating cutting temperature and thermal effects over the whole hybrid structure.

## 1. Introduction

Fibre reinforced polymers (FRP) have become increasingly popular in the past several decades because of their attractive mechanical performances, yielding remarkable lightening of systems’ masses and, hence, substantial reductions in energy consumption [1,2]. Nevertheless, conventional FRP suffers from heat resistance under critical temperature and their machining remains challenging in several applications. Unfortunately, research still mostly focuses on the influence of cutting tool specifications and fibre orientation in orthogonal cutting outputs while heat generation effects remain understudied. In particular, the curing cycle was found to have no effect on damage induced [3]. 

Hybrid composites associating carbon fibre reinforced polymers (CFRP) with titanium (Ti) are designed to further enhance thermomechanical compliance and overcome the issues encountered when cutting complex parts for aerospace and aeronautic sectors [4]. Typically, drilling is widely conducted on such structures for the preparation of holes that are crucial to processes like fastening e.g., riveting, bolting, etc. Different researchers have indicated that serious damage—such as poor hole surface quality, significant diameter error, delamination, fibre burrs, and polymer matrix degradation—can occur during drilling of CFRP/Ti stacks. The cutting speed, feed rate, drill bit geometry, cooling conditions, and stack sequences are the main parameters that affect the drilling temperature within both the CFRP and Ti phases as reported by An et al. [5]. The wide difference in the machinability of constituent parts of composite stacks is the result of gaps in cutting forces and chip formation mechanisms observed within both FRP and Ti phases. This incurs serious discontinuities along the CFRP/Ti interface at the instant of tool passage from one phase to the other, enforcing damage progression, and hence, complicating the control of material removal process. In drilling, it appears that these discontinuities depend sensitively on stacking order [6,7,8]. 

Isbilir et al. [7] omitted the temperature effects when investigating the drilling of CFRP and Ti, separately, as well as CFRP/Ti stack. The thrust force, torque, and the average surface roughness typically increase. When drilling stack structure, delamination was found to be less critical than when drilling CFRP singly. However, the tool exhibited significant life drop in drilling stack structure compared to its life in drilling CFRP or Ti separately because of the interaction of wear mechanisms in the two constituents within CFRP/Ti stack. An et al. [8] studied the effects of drilling strategies on static mechanical property and fatigue behavior of CFRP/Ti stacks, however passed over the resulting thermal effects in the drilling step. Unfortunately, thermal analyses mostly remain restricted to conventional FRP and pure experimental approaches [9,10,11,12,13,14,15,16,17].

Fundamentally, heat partition is a critical factor that affects the surface integrity and structure properties when cutting CFRP, Ti or CFRP/Ti stacks. Heat generation is sensitively governed by the anisotropy of structure and conductivities of the constituents in FRP. However, it is mostly affected by process parameters when cutting titanium [18,19,20,21]. The impact of cutting temperature appears more critical when cutting fibre metal laminates or composite-metal stacks. The behavior of hybrid composites is complex and it enhances heat discontinuities at the interface region especially in drilling. Ramulu et al. [22] carried out drilling tests on Graphite/Bismaleimide (Gr/Bi)/Ti stacks to assess cutting efficiency of three High Speed Steel (HSS) tools. The authors addressed the influence of feed, speed, and drill bit type on the hole quality, tool wear, and cutting forces. It appears that critical damage localizes close to the hole at the interface of Gr/Bi when using HSS-Cobalt and HSS drills while Tungsten–Carbide (WC) tool exhibits better tool life and involves the lowest surface and heat-induced damages. However, Wang et al. [23] studied the effects of low-temperature air on the performance of drilling CFRP/Ti stacks. Experimental findings showed that low-temperature air effectively reduces the thrust force and tool wear. In same vein, Wei et al. [24] investigated the effects of tool geometry on the drilling performance of CFRP/Ti stacks at real-time temperature, using thermocouples pre-installed into drills. In the CFRP phase, drilling temperature was found to decrease with feed rate increase but was insensitive to cutting speed variation. Nevertheless, in the Ti phase, temperature increases with elevation of both feed rate and cutting speed. The results revealed that the multi-facet drill yielded a lower temperature compared to the brad spur drill. When examining drilling temperature in CFRP/Ti stacks, Jia et al. [25] proved that peak value was reached at the interface region. Fibre orientation and stacking sequence play the most significant role in structure integrity at interface. Research has also apprehended the temperature analysis in drilling CFRP/Al stacks [26,27,28] and GLARE laminates [29]. Moreover, Castillo-Morales et al. [30] investigated the temperature distribution owing to edge trimming of CFRP/Ti6Al4V stacks using thermocouples embedded within the tool and workpiece. The feed rate is the most significant factor affecting the cutting temperature. Ge et al. [31] performed helical milling tests on CFRP/Ti stacks with sustainable cooling/lubrication (dry, MQL and cryogenic −167 °C to −94 °C) conditions. Temperature was especially analyzed at the CFRP layer, Ti-6Al-4V layer, and at the interface. 

Because it offers the advantage of understanding elementary mechanisms, orthogonal cutting assumption is widely applied in thermomechanical modeling of conventional FRP machining e.g., [9,10,11,12,14,15,32,33,34,35]. However, significant deficiencies in thermomechanical modeling of new hybrid composites still exist. Nowadays, all studies dealing with the modeling of CFRP/Ti composites omit temperature effects e.g., [4,36,37]. A flagrant lack of work on machining-induced thermal damage was detected in open literature. 

The present contribution proposes a damage-coupled heat finite element model to forecast the effects of drilling-induced temperature on constituting phases of hybrid composites. A finite sequence of drilling steps was simulated based on orthogonal cutting assumptions. To achieve this aim, we implemented a user-material subroutine VUMAT, communicating incrementally with a user-defined subroutine VDFLUX, into an ABAQUS/Explicit finite element commercial code. Among other outputs, temperature history and thermal damage owing to drilling were especially discussed. The reliability of the approach was highlighted by comparing the predictions to experimental data. 

## 2. Materials and Methods

### 2.1. Heat Dissipated

**Figure 1:**
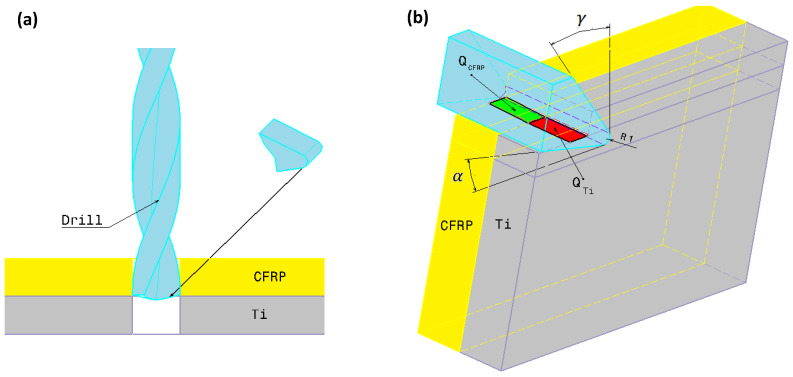
(**a**) Drilling model, (**b**) Thermomechanical model.

In orthogonal cutting of hybrid CFRP/Ti, the majority of the energy consumed is converted to heat. If the heat quantity absorbed by the tool and the chip is neglected, one can assume that the heat quantity ($Q_{w}$) flowing into the workpiece is given as follows:

(1) $$Q_{w}=Q_{CFRP}+Q_{Ti}$$

where $Q_{CFRP}$ is the heat dissipated by the composite phase, and $Q_{Ti}$ is the heat dissipated by the titanium phase. Figure 1a presents the drilling operation and the transformation of the 3D problem to the orthogonal cutting configuration Figure 1b. The heat fluxes at the tool/CFRP interface and the tool/Ti interface is assumed to be different because of the discrepancy in friction generated at the contact area of each phase. 

The total mechanical energy developed by the effect of cutting speed $\left(v_{c}\right)$ and the cutting force $\left(F_{c}\right)$ in the primary shear zone is,

(2) $$W_{P}=F_{c}v_{c}$$

The energy developed in the secondary shear zone by the effects of friction at tool-material interface is,

(3) $$W_{S}=\frac{F_{fr}v_{c}}{r_{c}}$$

where $F_{fr}=F_{c}sin\gamma +F_{t}cos\gamma$ is the total shear force acting on the tool rake face, γ the tool rake angle, and $r_{c}$ the chip compression ratio. 

It was widely accepted that all mechanical work done in the cutting process is converted into heat. Hence, the total heat generation $Q_{c}$ may be calculated from the cutting work as,

(4) $$Q_{c}=W_{P}+W_{S}=F_{c}v_{c}+\frac{F_{fr}v_{c}}{r_{c}}$$

or,

(5) $$Q_{w}+Q_{tool}^{P}+Q_{tool}^{S}+Q_{chip}=F_{c}v_{c}+\frac{F_{fr}v_{c}}{r_{c}}$$

where $Q_{w}$ is the heat resulting in plastic deformation within the primary shear zone, $Q_{chip}$ is the cutting heat generated in the chip, owing to plastic deformation and friction in the secondary shear zone, and $Q_{tool}^{P}$ and $Q_{tool}^{S}$ are the cutting heat transferred to the tool by the effect of localization in the primary and secondary shear zones, respectively.

Here, our focus will be mainly on the heat flowing into the workpiece, which highlights the effects of thermomechanical interaction on the integrity of the hybrid structure. Hence, only the $Q_{w}$ quantity could be implemented into the ABAQUS/Explicit commercial code, assuming localization in the primary shear zone has negligible effects. Thus, Equation (4) reduces to:

(6) $$Q_{w}+Q_{tool}^{P}=F_{c}v_{c}$$

It was widely accepted that heat localization in the secondary shear zone is the main cause of the heat ratio flowing to the tool body. Hence, Equation (6) can be further simplified to,

(7) $$Q_{w}=F_{c}v_{c}$$

For these calculations, the total energy was estimated based on the experimental results.

### 2.2. Estimation of Heat Flux Applied to CFRP

**Figure 2:**
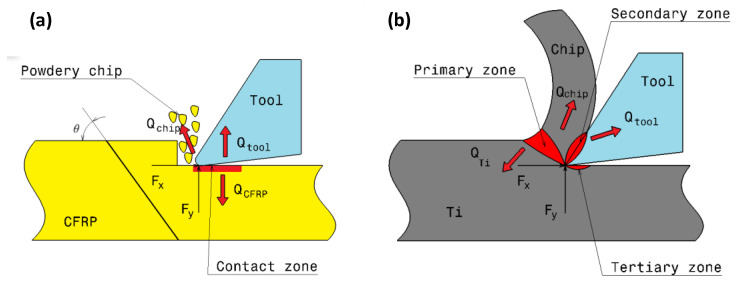
Heat partition models in (**a**) CFRP, and in (**b**) Titanium.

The anisotropy and inhomogeneity of unidirectional CFRP composites lead to different properties in different directions. Implicitly, this suggests that the greatest quantity of heat flows into the workpiece sensitively to the fibre orientation as the friction at tool-workpiece contact area varies with θ (Figure 2a). This results in the variation of the cutting force appearing in the heat flux equation. In orthogonal cutting of CFRPs, the heat flux, $q_{CFRP}$, applied to the workpiece is governed by,

(8) $$q_{CFRP}=\lambda_{CFRP}\frac{{F_{c}}_{CFRP}\times v_{c}}{A_{CFRP}}$$

where $\lambda_{CFRP}$ is the partition ratio of the heat energy that flows into the workpiece [11], ${F_{c}}_{CFRP}$ the horizontal cutting force, and $A_{CFRP}$ the contact area between the composite workpiece and the moving tool as given by,

(9) ACFRP=90°+α0+γ0180°πRc+1sin⁡α0·3Ft4E*Rc23·tCFRP

where $\alpha_{0}$ is the tool clearance angle, $\gamma_{0}$ the tool rake angle, $R_{c}$ the tool edge radius, $F_{t}$ the thrust force, $t_{CFRP}$ the thickness of the CFRP phase, $E^{*}$ the equivalent elastic modulus of the cutting tool and the CFRP phase as given by,

(10) $$\frac{1}{E^{*}}=\left(\frac{1}{G_{12}}-2\frac{\upsilon_{21}}{E_{11}}\right)\cdot \sin^{2}\theta \cos^{2}\theta +\frac{(1-\upsilon_{12}^{2})\sin^{4}\theta }{E_{11}}+\frac{\cos^{4}\theta }{E_{22}}+\frac{1-\upsilon^{2}}{E}$$

where θ is the fibre orientation, $G_{12}$ the shear modulus of the composite phase, $E_{ii}$ the elastic modulus of the composite phase in *i* direction, $\upsilon_{ij}$ the Poisson’s ratio of the composite phase for transverse strain in the *j* direction when the stress is in the *i* direction, and υ and E are the Poisson’s ratio and the elastic modulus of the tool, respectively.

### 2.3. Estimation of Heat Flux Applied to Titanium

In the orthogonal cutting process of metals, the heat is distributed over primary, secondary, and tertiary shear zones, as shown in Figure 2a. However, most contributions restrict the heat localization to primary and secondary deformation zones [19,38,39,40]. Referring to a literature review, it appears that the heat partition ratios flowing into the cutting tool, the workpiece, and the chip range between 0.02–0.18, 0.01–0.2, and 0.74–0.96, respectively [19,38].

In the proposed model, the tool body is considered analytically rigid. Indeed, it is assumed that it dissipates no heat at its flank face in contact with the formed chip. In addition, the continuous titanium chip forms simultaneously with the composite chip. As the former is powdery, the heat exchange between the formed chips within the interface in the secondary shear zone was neglected, and the heat affecting the interface was assumed to be flowing from the primary shear zone. Only that quantity was described in ABAQUS/Explicit code. This heat quantity denoted here as $q_{Ti}$ may be calculated from the developed work owing to the cutting force, as formulated in Equation (11),

(11) $$q_{Ti}=\lambda_{Ti}\frac{{F_{c}}_{Ti}\times v_{c}}{A_{Ti}}$$

where $\lambda_{Ti}$ denotes the partition ratio of the heat energy that flows into the workpiece. In the present study, $\lambda_{Ti}$ is fixed to 0.2 [19,38]. Meanwhile ${F_{c}}_{Ti}$ is the cutting force and $A_{Ti}$ is the effective area of titanium phase at the primary shear zone.

## 3. Constitutive Approach

### 3.1. Heat Transfer Model for Hybrid Composite 

The transient heat transfer process in the orthogonal cutting of hybrid CFRP/Ti composite is formulated according to the heat conduction equation, written as follows:

(12) $$\lambda_{11}\frac{\partial^{2}T}{\partial x^{2}}+\lambda_{22}\frac{\partial^{2}T}{\partial y^{2}}+\lambda_{33}\frac{\partial^{2}T}{\partial z^{2}}+q(x,y,z)=\rho c\frac{\partial T}{\partial t}$$

where $\lambda_{11}$ is the thermal conductivity parallel to the fibre direction, $\lambda_{22}$ and $\lambda_{33}$ are the thermal conductivities in transverse directions, q is the heat flux, and T is the relative temperature rise.

### 3.2. CFRP Constitutive Behavior

#### 3.2.1. Temperature-Dependent Properties

As we have seen, the thermo-physical properties of unidirectional CFRP composites are sensitive to elevation of temperature owing to cutting actions. Thus, the model of Gibson et al. [41], which is based on hyperbolic tangent function, is considered as a means of keeping the composite properties updated during machining. The aforementioned model is described as:

(13) $$P\left(T\right)=\frac{1}{2}\left(P_{0}-P_{r}\right)tanh\left[-\frac{1}{∆T/2}\left(T-\left(T_{g}+\frac{∆T}{2}\right)\right)\right]+\frac{1}{2}\left(P_{0}+P_{r}\right)$$

Here, P denotes the mechanical property at a given temperature T, $P_{0}$ denotes the mechanical property at room temperature, and $P_{r}$ denotes the relaxed (high temperature) value of the mechanical properties. $T_{g}$ is the glass transition temperature, and ∆T the temperature variation from the glass transition point of the polymer matrix.

#### 3.2.2. Damage Initiation Criteria

Damage initiation in the composite phase refers to the onset of degradation at a material point. In unidirectional fibre composites, the damage can be predicted using various failure criteria e.g., stress or strain criteria, Tsai-Hill criteria, and Tsai-Wu criteria [42]. However, the most famous one is Hashin’s criteria, which distinguishes the appropriate damage mode for each phase in the laminate and for each loading mode. Thus, each failure criterion is sensitive to developed stresses. At a given material point, the material properties degrade according to the required failure mode once these criteria are reached. Hence, the stresses at the target area update to reflect the properties’ degradation according to the failure mode. The composite behavior is assumed to be linear elastic up until the point of failure. The failure criteria are reported in Table 1.

In the above equations, $\sigma_{ii\left(i=1,2,3\right)}$ and $\tau_{ij\left(i\neq j;i,j=1,2,3\right)}$ represents the effective stress tensor, $X_{T}$ and $Y_{T}$ the tensile failure stresses in longitudinal and transverse directions, respectively, $X_{C}$ and $Y_{C}$ the compressive failure stresses in longitudinal and transverse directions, respectively, $S_{ij(i\#j;i,j=1,2,3)}$ the components of the in-plane and out-of plane shear failure stresses.

#### 3.2.3. Damage Evolution

The damage progression is controlled by an exponential law referring to the Matzenmiller et al. [43] function. The fibre and matrix damage variable can be expressed as per the following:

(14) $$d_{i}^{j}=1-exp\left(\frac{1}{m_{i}^{j}}{\left(1-R_{i}\right)}^{m_{i}^{j}}\right),i\in \left[1t,1c,2t,2c\right]$$

where $m_{i,i=t,c}^{j,j=f,m}$ are the material softening parameters that control the rate of degradation for each loading mode. According to Khan et al. [42], the coefficients $m_{t}^{f}$, $m_{c}^{f}$, $m_{t}^{m}$, and $m_{c}^{m}$ used in the proposed model were calibrated to 0.2, 0.07, 1.15, and 3, respectively.

### 3.3. Titanium Constitutive Behavior

For the titanium phase, the Johnson–Cook constitutive model and damage criteria [44,45] are introduced to describe the high plastic deformation, and strain rate in close relation with temperature evolution during the cutting operation. The equivalent flow stress is calculated based on material constants as following:

(15) $$\bar{\sigma }=\left[A+B{\left(\bar{\varepsilon }\right)}^{n}\right]\left[1+Cln\left(\frac{\dot{\bar{\varepsilon }}}{{\dot{\bar{\varepsilon }}}_{0}}\right)\right]\left[1-{\left(\frac{T-T_{0}}{T_{m}-T_{0}}\right)}^{m}\right]$$

where $\bar{\sigma }$ is the equivalent flow stress, $\bar{\varepsilon }$ the equivalent plastic strain, $\dot{\bar{\varepsilon }}$ the equivalent plastic strain rate, ${\dot{\bar{\varepsilon }}}_{0}$ the reference equivalent plastic strain rate, T the effective temperature reached in the titanium phase, $T_{m}$ the material melting temperature, and $T_{0}$ the room temperature. A, B, C, m, and n are the material constants to be determined experimentally. The Johnson– Cook damage criteria is also implemented into the titanium phase to simulate the material removal process. The equivalent plastic strain at damage initiation [44,45] is described as shown in Equation (16),

(16) $${\bar{\varepsilon }}_{0}=\left[d_{1}+d_{2}exp\left(d_{3}\frac{P}{\bar{\sigma }}\right)\right]\left[1+d_{4}ln\left(\frac{\dot{\bar{\varepsilon }}}{{\dot{\bar{\varepsilon }}}_{0}}\right)\right]\left[1+d_{5}\left(\frac{T-T_{0}}{T_{m}-T_{0}}\right)\right]$$

where ${\bar{\varepsilon }}_{0}$ is the plastic strain at damage initiation, P the hydrostatic pressure, $d_{i,i=1\ldots 5}$ the failure constants to be obtained experimentally with notched and axisymmetric specimens at different stress state, temperatures, and strain rate. Thus, the chip separation occurs when ${\bar{\varepsilon }}_{0}$ reaches the plastic strain threshold, and the fracture criteria defined below, reaches one (*f* = 1):

(17) $$f=\frac{\sum \bar{\varepsilon }}{{\bar{\varepsilon }}_{0}}$$

All numerical inputs are carefully specified for describing the Johnson–Cook constitutive model and damage model. Preliminary simulations were initially conducted to simulate the cutting process of titanium phase singly and the ability of the user-defined material to predict the chip formation mechanisms was well proven.

### 3.4. Finite Element Model

A 3D damage model is implemented into the finite element code through a user-defined subroutine VUMAT available upon the ABAQUS/Explicit code. This allows us to predict the damage extent and mode within each of the phases of the hybrid structure depending on the type of loading within the target finite element. The damage modeling uses full 3D stress state. The temperature-coupled displacement approach was implemented by a user-defined VDFLUX, which acts to sensitively update the material properties by referring to temperature increments. The efficiency of temperature-coupled displacement was firstly investigated in a simulating tensile test. Then, the modeling addressed the cutting of CFRP composite singly to prove the reliability of the damage-coupled elasticity approach as described by the VUMAT. A moving surface heat flux applied at the trim plane, and at the tool-material interface, is applied to simulate the source of cutting-generated temperature. Predictions owing to the cutting of the CFRP phase singly were discussed and outputs were compared to experimental findings. Finally, the reliability of the proposed model to simulate the cutting of hybrids i.e., CFRP/Ti was discussed with reference to both the predicted and measured outputs. Thus, validation of the proposed approach involves the investigation of the tensile test, the cutting process of CFRP phase singly, and the cutting process of CFRP/Ti hybrid composite. The geometry of the cutting models is reported in Table 2 together with the mesh structure, type, and size.

## 4. Results and Discussion

### 4.1. Reliability of Thermomechanical Approach in Tensile Test Simulation

#### 4.1.1. Tensile Test Model

In order to validate the temperature-coupled damage approach, the uniaxial tensile test of the CFRP composite was simulated with reference to the experimental data available in [46]. The mechanical properties implemented for calculations are summarized in Table 3.

**Figure 3:**
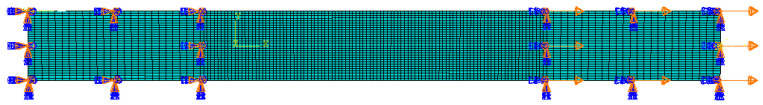
FE model built for tensile test simulation showing mesh type and boundary conditions.

A 3D tensile test specimen of 200 × 20 × 1.35 mm^3^ [47] was used for strength and failure process simulation in longitudinal direction. The mesh uses an integral solid element reduced to eight nodes that are thermally coupled (C3D8T). The loading and boundary conditions were in concordance with the real testing conditions. Axial displacement was applied on one end of the specimen while the other end was kept fixed (Figure 3).

#### 4.1.2. Analysis of Tensile Test Outputs vs. Temperature

**Figure 4:**
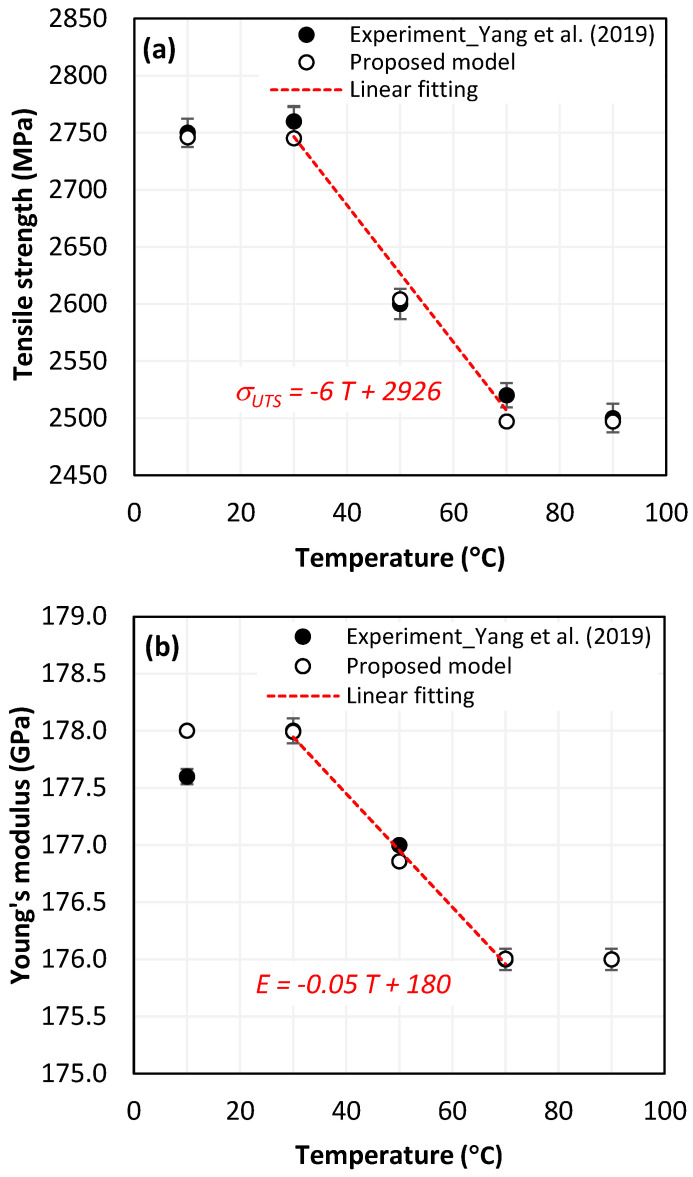
(**a**) Tensile strength vs. temperature [1], and (**b**) Young’s modulus vs. temperature [1].

Tensile test simulation of CFRP singly has been introduced in [48]. Preliminary results obtained revealed the sensitivity of outputs to temperature variation. Figure 4a presents the predicted versus measured tensile strength when the temperature varies. The predictions show a good agreement with the experimental findings since the maximum gap between results does not exceed 1% (at 70 °C). A severe linear degradation of the property was observed especially within the range of 30–70 °C. Obviously, at low temperatures (T ≤ 30 °C) approximating $T_{0}$, the tensile strength evolution remains invisible. When temperature approaches glass transition point (T > 70°C), the material loses its integrity balance, and the dependency of its behavior in relation to temperature substantially attenuates.

Figure 4b shows the variations of elastic modulus of CFRP versus temperature. The predicted the elastic modulus at 10 °C i.e., 178 GPa exhibits the highest discrepancy of about 0.22% compared to the measured one that approximates 177.6 GPa while predictions at temperatures over 10 °C present insignificant errors if compared to measurements. This reflects the reliability of the FE model to estimate the elastic modulus. By referring to the value calculated at 10 °C, the elastic modulus predicted at higher temperatures drops by approximately 0.005%, 0.64%, 1.12% and 1.12%, respectively.

It is worth noting that the critical degradation rates of the tensile properties were all recorded within 30–70 °C. While the glass transition temperature of Epoxy resin generally ranges between 55 and 120 °C, it appears that damage initiates even before reaching T_g_ value. This is attributed especially to the discontinuity along the fibre-matrix interfaces, which acts to promote failure to initiate at relatively low testing temperature.

#### 4.1.3. Failure Modes vs. Temperature

Without doubt, the failure aspect depends on effective temperature during testing. Table 4 reports typical modes of failure obtained at extreme temperatures applied when loading unidirectional CFRP specimens longitudinally.

At relatively low temperature, i.e., 10 °C the resin resists the uniaxial loading together with fibres because of the fibre-matrix interface adhesion. Thus, the composite failure yields fragments of those fibres covered by the resin matrix. Mode I failure dominates in the longitudinal direction, enhancing matrix cracking especially at the ply-to-ply plane. Separated ply segments undergo transverse Mode-II failure in series up until the final fracture of the specimen. However, at a high temperature level, e.g., 90 °C, the matrix resistance drops drastically, resulting first in fibre-matrix interface failure. Physically, the uniaxial loading is resisted mostly by the uncovered fibre units provided that their properties have not been unaffected by the heat generated. In this case, Mode-I is quasi-totally discarded and failure is governed by premature Mode-II acting up until total fracture. The simulation reliably reproduces the failure modes across acting versus applied temperatures. At high testing temperatures, the von Mises stress fields inform about the damage affected area, reflecting the failure modes dominating the CFRP behavior.

### 4.2. Reliability of Thermomechanical Approach in Cutting CFRP Singly

#### 4.2.1. Cutting Model

The cutting model was built as described by Qian et al. [12]. A constant heat flux was applied at the top surface of the trim plane to simulate the temperature generation source (Table 2). The heat flux locates along the tool material interface and moves as the tool advances. The mesh was constructed with eight-node thermally-coupled brick elements (C3D8T) available in ABAQUS/Explicit code. The mesh within the refined region uses finite element of 2.5 μm in size. In this case, the friction coefficient varies with fibre orientation as contact conditions between the tool and composite phases govern the material removal process, deciding which of the mechanisms dominate the chip formation. 

The Coulomb friction law is applied to update the frictional stress at the tool-composite interfaces, as described by,

(18) $$\tau_{f}=\mu \left(\theta \right)\tau_{n}$$

where $\tau_{f}$ and $\tau_{n}$ are the frictional and normal stresses, respectively, and μ is the friction coefficient varying with fibre orientation. The values of μ considered in the simulation of cutting process are based on orthogonal cutting tests conducted on unidirectional composite structures [12,49]. The material properties [11] and cutting conditions, including friction coefficients, [12] are listed in Table 5.

#### 4.2.2. Cutting Temperature vs. Fibre Orientation

**Figure 5:**
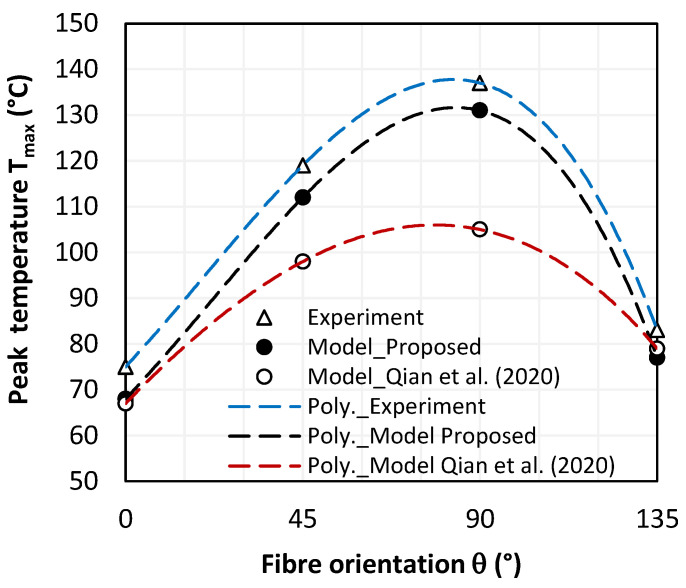
Peak values of cutting temperature vs. fibre orientation [12].

Figure 5 compares the experimental and numerical results of an orthogonal cutting process of CFRP plate. Roughly, the proposed approach leads to reliable results since predicted values of cutting temperature look in good agreement with the cutting test results presented by Qian et al. [12]. Compared with experiments, the predictions yield an average gap of about 6.5 °C which does not exceed 9.3% in the most unfavorable case i.e., θ = 0°.

For a 90° fibre orientation, we can see that the cutting temperature reaches its maximum. This is mainly attributed to the fact that when the fibres are being cut transversely, they generate a large contact area with the clearance face of the tool. As fibres are poor thermal conductors, temperature localizes at their cut cross area which yields a relatively high temperature field while the highest friction coefficient is measured at θ = 135°. This proves that chip formation results in several complex elementary mechanisms i.e., friction, failure mode, damage progression mode, etc. that can act in combination for deciding the chip’s formation and composite structure integrity both at and close to the 90°-fibre orientation.

Since CFRP is a poor thermal conductor, the induced heat does not dissipate enough into the surrounding environment during the cutting period, which yields localized damages resulting in material property degradation. Typically, critical damage occurs when temperature exceeds the glass transition point of the resin. From the plots, Qian et al.’s micromechanical model underestimates the generated temperature when compared to the experimental data and proposed model predictions, which both capture critical values close to 90°, while T_g_ approximates 120 °C.

### 4.3. Reliability of Thermomechanical Approach When Cutting CFRP/Ti Hybrids

#### 4.3.1. Numerical Model: Cutting and Boundary Conditions

It is worth noting that the total heat generated by machining is calculated based on available experimental data and the heat ratio to be flowed into the workpiece is used as input for the model. The model aims at predicting the effects of temperature distribution on the structure integrity of CFRP/Ti6Al4V. The properties of CFRP phase required for the user-defined subroutines are cited in Table 5 while the parameters implemented according to the Johnson–Cook constitutive model (Equation (14)) and damage model (Equation (15)) are reported in Table 6, as well as the mechanical properties of the titanium alloy.

In order to account for the sensitive change in temperature history with cutting parameters, the DOE for simulations involves variation of cutting speed and fibre orientations. All the runs are conducted on commercial finite element code ABAQUS/Explicit. In order to account for the sensitive change in temperature history with cutting parameters, the DOE for simulations involves variation of cutting speed and fibre orientations.

All the runs are conducted on commercial finite element code ABAQUS/Explicit. The tool specifications, workpiece geometry, and cutting parameters are reported in Table 7. The workpiece was modeled as a prismatic part, including both titanium and CFRP phases of equal width ($w_{CFRP}=w_{Ti}=0.4\;mm$). The tool modeled as a fully rigid body is only free in the cutting direction while all the degrees of freedom were completely constrained at the bottom surface of the workpiece. The contact interaction between the stacked material and the tool is managed by the algorithm’s surface-to-node surface contact, available in ABAQUS/Explicit code (and based on the friction coefficients obtained experimentally in [12] and specified in Table 7).

The model does not consider the interfacial layer since the stack constituents are only in contact during actual processing. The “Tie” property is used to describe the interaction condition between the stack phases. The mesh of the workpiece was refined in the cutting zone using a finite element size of 40 μm and, as when modeling the cutting of CFRP singly, the mesh uses eight-node thermally coupled displacement brick elements (C3D8T) that allow for temperature capture during the cutting process.

#### 4.3.2. Sensitivity of Chip Formation to Heat Transfer within Phases

In the orthogonal cutting process of CFRP/Ti stack, the thermomechanical interaction between the Ti phase and the CFRP phase is evident, compared with cutting CFRP singly. This has a significant influence on temperature within the interface. As shown in Table 8, under the same tool specifications and cutting parameters, the temperature in CFRP phase appears significantly affected by the change in fibre orientation. The cutting temperature of CFRP plate reaches its peak value systematically at the interface irrespective of the fibre orientation. The exposure of free surface of the CFRP favors heat loss and thus leads to temperature values that are lower compared with those at interface. However, the titanium phase exhibits a quasi-uniform temperature field within the thickness. The gap between the values obtained at free surface and at interface is negligible compared to that recorded between the CFRP surfaces, respectively. Simulation also reproduces the chip formation mechanisms through the hybrid constituents since it predicts reliably both continuous and fragmented chips resulting in Ti and CFRP phases, respectively.

The chip generated from the CFRP phase does not appear because the “element deletion” option was used to simulate the material removal process when any of the damage criteria defined above (Table 1) were reached. However, the cutting process yields a continuous chip from the Ti phase which fits with the physical mechanisms widely observed when cutting titanium [17,18,19,20]. When investigating the predicted fields at the CFRP interface side, it can be pointed out that even after tool passage, temperature still localizes at relatively high values along the trim plane-to-interface common edge. In fact, the heat quantity generated at the Ti volume acts as a heat source to keep temperature at the frontier as high as possible while the CFRP free surface seems to favor heat loss and create unbalanced temperature fields through the width direction with increased localization towards the interface, regardless of the fibre orientation. Such localization acts to involve thermal discontinuities within the interface, leading to permanent change in phases’ properties and, thus, to undesired behavior of the stack structure.

#### 4.3.3. Temperature Measurement

**Figure 6:**
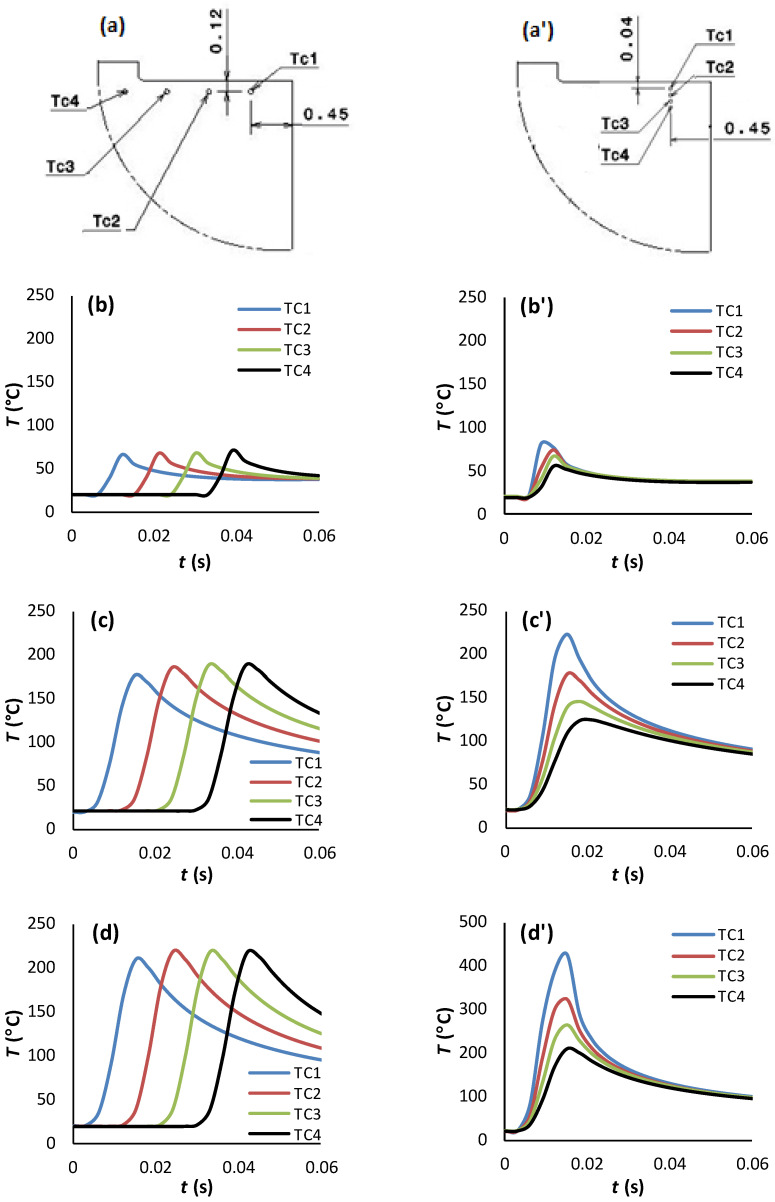
Temperature history captured when cutting CFRP/Ti ($\upsilon_{c}=50\;mms^{-1}$, θ=90°). (**a**,**a’**) TCs location in-front and in-depth directions, respectively. (**b**–**d**) In-front temperature vs. time recorded at CFRP, interface, and Ti phases, respectively. (**b’**–**d’**) In-depth temperature vs. time recorded at CFRP, interface, and Ti phases, respectively.

Figure 6 reports the temperature history generated within the subsurface of the hybrid constituents. Analysis was intentionally restricted to the case of *θ* = 90° since it is the more likely orientation for the highest in-depth temperature dissipation. 

**Figure 7:**
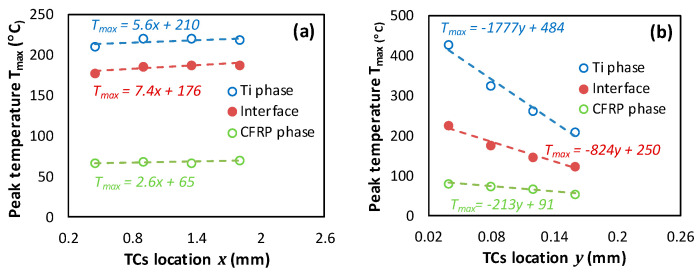
Peak temperature captured at TCs location ($\upsilon_{c}=50\;mm\;s^{-1}$, θ=90°). (**a**) In-front direction, (**b**) In-depth direction.

TCs are intentionally located within the subsurface in-front and in-depth directions to capture temperature at interface, CFRP, and Ti free surfaces. To avoid the effects of boundary conditions, TCs were located 0.45 mm away from the reference face—from which cutting of the stack starts. Roughly, temperature increases from the CFRP free surface towards the Ti free surface mark intermediate values at the interface regardless of the measurement direction. Based on these numerical outputs, the following points can be drawn:

- Referring to Figure 6b–d, when the tool advances from TC1 to TC4, the temperature at CFRP, interface, and titanium phases rises by 6.03%, 5.86%, and 3.94%, respectively by the effect of heat flow accumulation brought by the cutting tool. These amounts look too close because of relatively small spacing between TCs locations.
- In contrast, when investigating the temperature fields’ in-depth direction as reported in Figure 6b’–d’, it was revealed that peak temperature drops when the depth increases. Thus, highest value was recorded at TC1, lowest value at TC4, and intermediate TCs record the values in-between. This can be rationally attributed to the heat localization that occurs along the nearest subsurface layers to trim plane. From predictions, peak temperature drops by 31.3%, 44.4% and 50.8% when passing from TC1 to TC4 at CFRP, interface, and titanium phases, respectively.
- In terms of in-front direction, predictions show that peak temperature increases with effective rates according to linear laws as $T_{max}({}^{\circ}C)=ax+b$ (Figure 7a), where x denotes the TCs location in-front direction. These laws are valuable out of the front and back free surfaces in which temperature depends sensitively on initial and boundary conditions. The surrounding environment acts to accelerate heat loss since external temperature i.e., room temperature, plays to favor heat dissipation through free surfaces. Both a and b are dependent material constants.
- In terms of in-depth direction, however, the peak temperature falls linearly with disparate rates following, $T_{max}\left({}^{\circ}C\right)=-\alpha y+\beta$ (Figure 7b), where α and β denote the law of dependent material constants, and y the TCs location in-depth direction. It can be outlined that peak values at the interface and Ti phase decrease ~4 and ~8 times faster than those captured at the CFRP phase while these ratios were found to fluctuate from ~2.8 to ~2.1, respectively, in the cutting direction. Although equal thermal conductivities, Ti phase seems to dissipate temperature much faster than CFRP because of the isotropic nature of the metallic phase, provided that transverse thermal conductivities of CFRP are both too low ($\left(\lambda_{22}=\lambda_{33}=0.8\;Wm^{-1}K^{-1}\right)$) compared to that in-plane ($\left(\lambda_{11}=7\;Wm^{-1}K^{-1}\right)$). Hence, the “*volume effect*” plays for dominating heat loss which explains the discrepancies between the temperature rates of the two constituents.

#### 4.3.4. Damage Contours Analysis

Thermal damage occurring at the critical localization area of temperature might be described as Heat Affected Zones (HAZ). In this area, material properties substantially fall until total failure. However, critical temperature values are sensitive to each phase since the properties of the two constituents of the stack are too different. 

**Figure 8:**
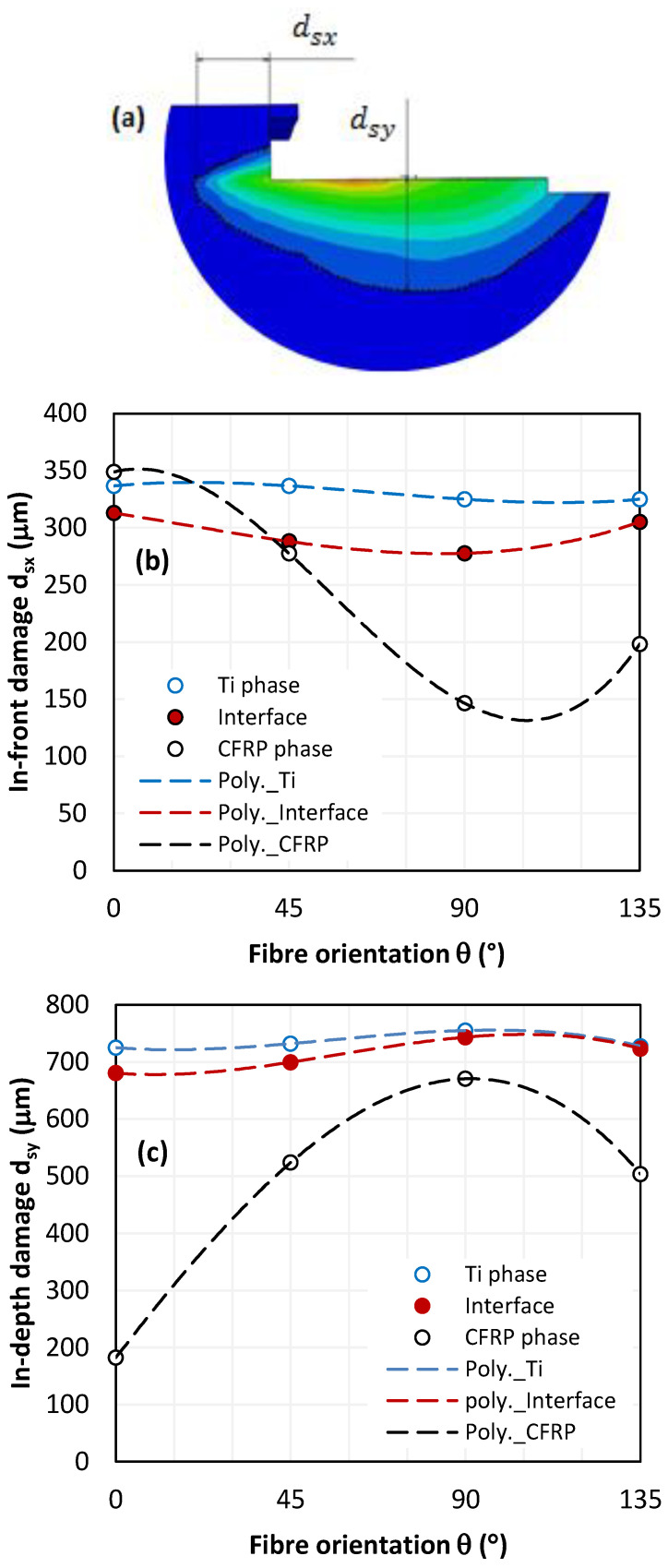
Temperature levels captured in-front and in-depth for the tool ($\upsilon_{c}=50\;mm\;s^{-1}$). (**a**) Damage measure description, (**b**) in-front damage, (**c**) in-depth damage.

Figure 8a shows the temperature contours involved due to cutting. The damage is, hence, defined as the most far temperature overlap from the trim plane limiting the heat affected zone. Both $d_{sx}$, and $d_{sy}$ describe the typical measures of damage in-front and in-depth to the tool, respectively. From simulations, it can be outlined that there are wide gaps between $d_{sx}$ and $d_{sy}$. Quasi-regular ratios were calculated at titanium and at interface while random fluctuations dominate the ratios between values predicted at CFRP phase.

It is worth noting that the temperature field is more oriented towards the subsurface since temperature overlaps neatly cover a larger affected area in-depth than in-front of the tool, except for CFRP with 0° oriented fibres. As expected, the deepest temperature within CFRP was captured at 90° fibre orientation because of relatively good conductivity in fibre direction i.e., $\lambda_{11}=7\;Wm^{-1}K^{-1}$. At CFRP/Ti interface, temperature contour depth fluctuates between the limits recorded within the Ti and CFRP constituents. However, it was found that temperature contour at the interface approximates much more than that obtained at titanium. The highest distance ratio (about 3.73) is calculated between the interface and CFRP for 0° fibre orientation.

Typically, the average ratio between damage values ($d_{sy}$/$d_{sx}$) approximates 2.2 ± 0.03 μm and 2.4 ± 0.09 μm at Ti phase and at interface, respectively (Figure 8b,c). In contrast, this ratio was found ranging from 0.2 to 1.9 μm for the CFRP phase which suggests that fibre orientation has a significant role in governing heat generation within the composite plate because of the sensitive variation of contact properties with fibre orienation. In fact, predictions show a damage variation that is quasi-stable at Ti and interface, resulting in a standard deviation that is relatively low if compared to that calculated over the values predicted at CFRP phase (Table 9).

Thus, the heat generated during material removal process seems to be highly dominated by the thermomechanical properties of CFRP phase i.e., fibre orientation, thermal conductivity evolution, etc., which decide the tool-material contact conditions, forces developed, and failure mode resulting in both the mechanical damage owing to cutting actions, and heat localization owing to friction at interfaces. Referring to the obtained results, the interface damage plot seems to locate between CFRP and Ti plots except for d_sx values obtained at 0° fibre orientation where the damage predicted at CFRP exceeds those recorded at interface and Ti. The relatively high thermal conductivity of CFRP in the longitudinal direction favors heat propagated in-front of the tool. However, d_sx values captured at CFRP and Ti remain too close since the proposed model assumes that in-plane longitudinal thermal conductivity of CFRP equals that of titanium, i.e., $\lambda_{11-CFRP}=\lambda_{Ti}=7\;Wm^{-1}K^{-1}$. 

Meanwhile, $d_{sx}\left(CFRP\right)$ at 0° fibre orientation overtakes the damage predicted at Ti phase by approximately 3.47%. This was mainly attributed to the isotropic nature of titanium which allows temperature dissipation at the same rate in all directions, while the heat flux in CFRP is rationally oriented—resulting in lower heat loss and, hence, a larger heat affected area. Although temperature at interface is logically ranging in the limits measured within the stack constituents, it exhibits a high sensitivity gap compared to the CFRP phase which results in thermal discontinuities that come to accentuate structural degradation along the interface during cutting. 

#### 4.3.5. Critical Thermal Damage

**Figure 9:**
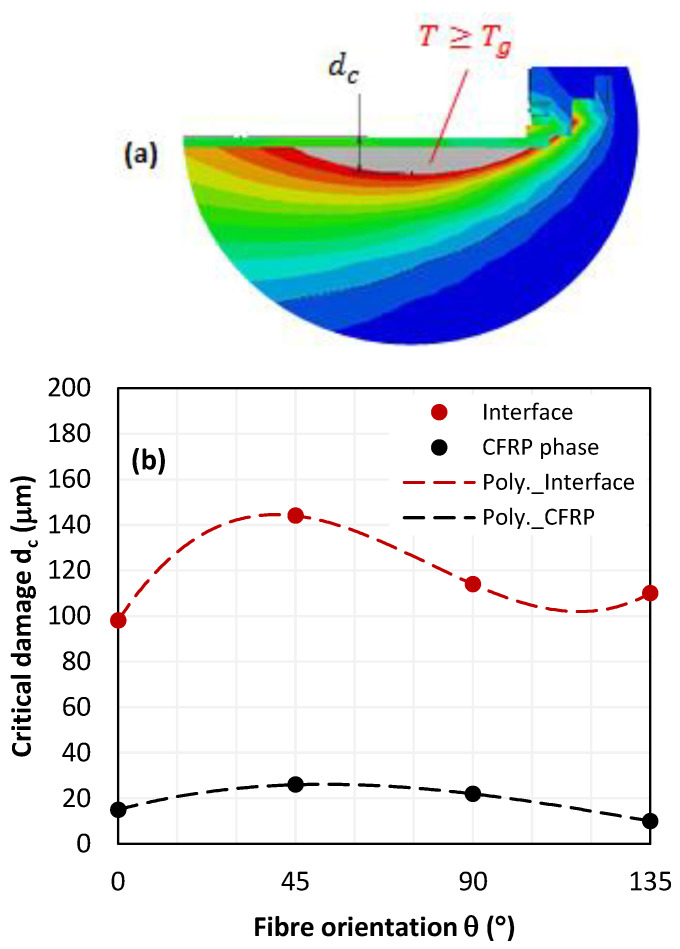
(**a**) Damage measure description, (**b**) in-depth temperature contour overlapping, $T\geq T_{g}$, captured at interface and at CFRP phase ($\upsilon_{c}=50\;mm\;s^{-1}$).

The influence of fibre orientation on thermal damage in interface and in CFRP plate was a particular focus of our investigation (Figure 9). Thermal damage varies sensitively with fibre orientation; however, a more pronounced fluctuation is obtained at interface. Unexpectedly, critical damage at interface does not occur at 90° fibre orientation.

At interface, the critically affected zone (T ≥ 150°C) is minimum for 0° orientation and the deepest overlap was captured for 45° fibre orientation. At CFRP, the predictions fluctuate with a standard deviation of about ±3 μm while such fluctuations reach ±8.5 μm at the interface, which is approximately three times higher than at the CFRP phase. 

**Figure 10:**
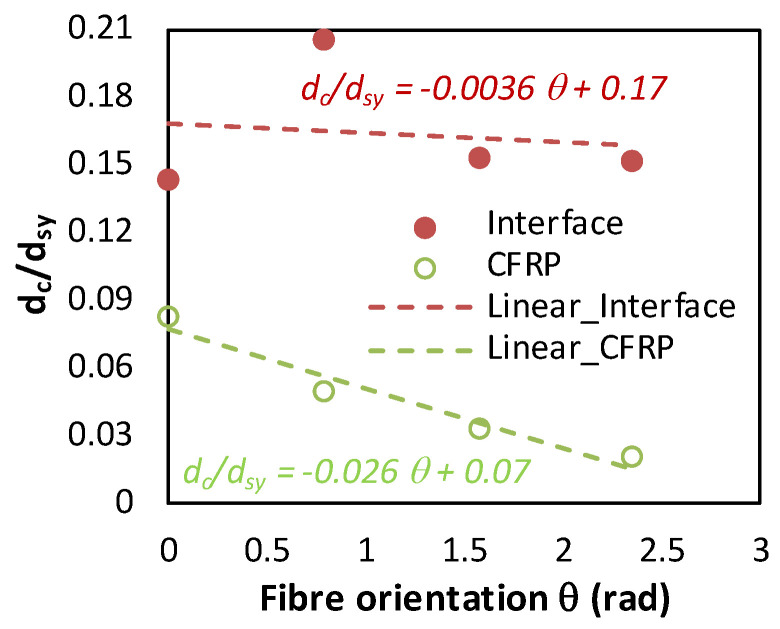
Dimensionless damage laws vs. fibre orientation at both interface and CFRP phase.

The discrepancies observed in the critically affected zone along the interface confirm the complexity of interacting mechanisms involved by effect of stacking. In fact, the in-depth damage state ($d_{sy}$) is directly related to critical damage $\left(d_{c}\right)$ since $\frac{d_{c}}{d_{sy}}$ the ratio is a linear function. Figure 10 reports the relationships $\frac{d_{c}}{d_{sy}}=f(\theta )$ at fixed cutting conditions while only fibre orientation varies.

The obtained fittings reveal an accelerated drop in the critical damage ratio at CFRP when θ increases. If compared with the interface, the damage ratio measured at CFRP decreases 7.2 times faster which signifies that hybrid behavior when cutting is significantly dominated by thermal state at CFRP phase.

## 5. Conclusions

This paper addresses a new thermomechanical approach for investigating the integrity of CFRP/Ti hybrid structures owing to single drilling shot. The study aims to understand thermal damage in order to better control the material removal process. In particular, it focuses on the effects of heat localization within the interface, Ti and CFRP phases. The reliability of the predictions was discussed through (i) the damage overlaps at composite phases, and (ii) the sensitivity of the interface to temperature generation. In light of the obtained results, the following concluding remarks can be drawn:

1. When cutting CFRP singly, fibre orientation was found to have significant effects on the cutting-induced temperature. The highest and lowest peak values were observed when cutting perpendicular and parallel to the fibre orientation, respectively. The current thermomechanical approach shows high reliability in predicting cutting-induced temperature when compared to Qian et al.’s experiments and model. The predictions-to-experiment errors range within 4.4–9.3% which confirms the efficiency of the proposed temperature-coupled displacement approach.
2. When investigating the most critical configuration of hybrid composite, i.e., 90° fibre orientation, the peak temperature drops linearly when the tool advances, regardless of the TCs locations. However, when it comes to the in-depth direction, the peak temperature at interface drops ~4 times lower than at CFRP phase. As for the in-front direction, this ratio does not exceed 2.8. However, temperature overlaps at the interface were found to be dominated by the temperature field generated within the Ti phase.
3. Damage analysis owing to temperature overlaps delimiting values higher than the glass transition point (T ≥ T_g_) shows severe localization at interface. Heat induced at the Ti phase seems to act together with opposite CFRP phase for enhancing temperature generation at interface. This involves severe discontinuity along the interface that seems to favor failure initiation and, hence, affects the hybrid structure’s integrity during cutting.

## Figures and Tables

**Table 1:** Hashin damage criteria considered for modeling failure in CFRP phase, singly [4].

Failure Mode	Hashin Criterion
Fibre tension $\sigma_{11}\geq 0$	${\left(\frac{\sigma_{11}}{X_{T}}\right)}^{2}+{\left(\frac{\tau_{12}}{S_{12}}\right)}^{2}+{\left(\frac{\tau_{13}}{S_{13}}\right)}^{2}=\left\{\begin{array}{l} R_{1t}\geq 1\;failure\\ R_{1t}<1\;nofailure \end{array}\right.$
Fibre compression $\sigma_{11}\leq 0$	${\left(\frac{\sigma_{11}}{X_{C}}\right)}^{2}=\left\{\begin{array}{l} R_{1c}\geq 1\;failure\\ R_{1c}<1\;nofailure \end{array}\right.$
Matrix cracking $\sigma_{22}+\sigma_{33}\geq 0$	$\frac{{\left(\sigma_{22}+\sigma_{33}\right)}^{2}}{{Y_{T}}^{2}}+\frac{{\tau_{23}}^{2}-\sigma_{22}\sigma_{33}}{{S_{23}}^{2}}+\frac{{\tau_{12}}^{2}+{\tau_{13}}^{2}}{{S_{12}}^{2}}=\left\{\begin{array}{l} R_{2t}\geq 1\;failure\\ R_{2t}<1\;nofailure \end{array}\right.$
Matrix compression $\sigma_{22}+\sigma_{33}\leq 0$	$\left[{\left(\frac{Y_{C}}{2S_{23}}\right)}^{2}-1\right]\frac{\left(\sigma_{22}+\sigma_{33}\right)}{Y_{C}}+\frac{{\left(\sigma_{22}+\sigma_{33}\right)}^{2}}{4S_{23}^{2}}+\frac{{\tau_{23}}^{2}-\sigma_{22}\sigma_{33}}{S_{23}^{2}}+\frac{{\tau_{12}}^{2}+{\tau_{13}}^{2}}{S_{12}^{2}}$ $=\left\{\begin{array}{l} R_{2c}\geq 1\;failure\\ R_{2c}<1\;nofailure \end{array}\right.$

**Table 2:** Model description considered for simulating cutting process mechanisms.

	Part Geometry	Model	Mesh Type
**CFRP**	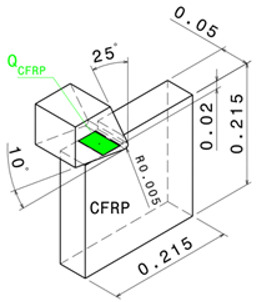	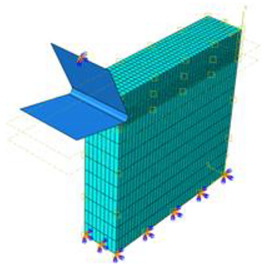	C3D8T, 2.5 μm
**CFRP/Ti**	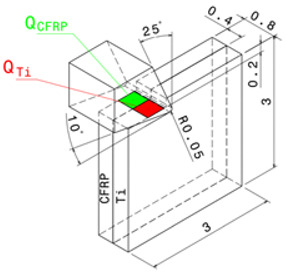	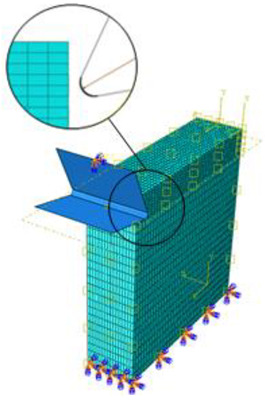	C3D8T, 40 μm

**Table 3:** Properties of CFRP used for tensile test simulation [46].

Parameters	Symbol	Value
Density	$\rho (Kgm^{-3})$	1600
Longitudinal Young’s modulus in-plane	$E_{11}(GPa)$	$178^{*}$
Transverse Young’s modulus in-plane	$E_{22}(GPa)$	9.1
Transverse Young’s modulus normal-to-plane	$E_{33}=E_{22}(GPa)$	9.1
In-plane shear modulus	$G_{12}(GPa)$	5.6
Shear modulus in plane 1–3	$G_{13}=G_{12}(GPa)$	5.6
Shear modulus in plane 2–3	$G_{23}(GPa)$	4
In plane Poisson’s ratio	$\nu_{12}$	0.31
Transverse Poisson’s ratio in 1–3 plane	$\nu_{13}=\nu_{12}$	0.31
Transverse Poisson’s ratio in 2–3 plane	$\nu_{23}$	0.45
Longitudinal tensile strength in-plane	$X_{t}(MPa)$	$2750^{*}$
Longitudinal compression strength in-plane	$X_{c}(MPa)$	1690
Transverse tensile strength in-plane	$Y_{t}(MPa)$	111
Transverse tensile strength normal-to-plane	$Z_{t}=Y_{t}(MPa)$	111
Transverse compression strength in-plane	$Y_{c}(MPa)$	214
Transverse compression strength normal-to-plane	$Z_{c}=Y_{c}(MPa)$	214
In-plane shear strength	$S_{12}(MPa)$	115
Transverse shear strength in plane 1–3	$S_{13}=S_{12}(MPa)$	115
Transverse shear strength in plane 2–3	$S_{23}(MPa)$	26
In-plane longitudinal thermal conductivity	$\lambda_{11}\left(Wm^{-1}K^{-1}\right)$	5
Transverse thermal conductivity in-plane	$\lambda_{22}\left(Wm^{-1}K^{-1}\right)$	0.42
Transverse thermal conductivity normal-to-plane	$\lambda_{33}\left(Wm^{-1}K^{-1}\right)$	0.42
Specific heat	$C_{p}\left(J{Kg}^{-1}K^{-1}\right)$	1040

* Yang et al. [1].

**Table 4:** Simulated and experimental failure modes obtained at 10 °C, and 90 °C, respectively [1].

T=10 °C	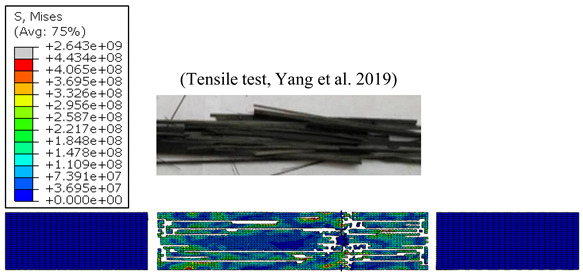
T=90 °C	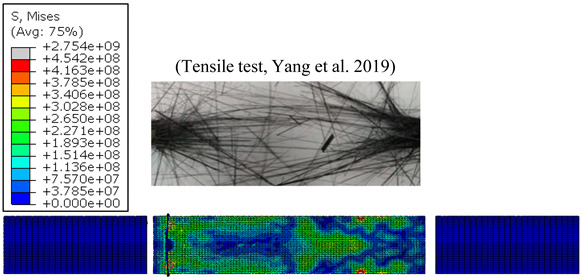

**Table 5:** Mechanical properties of CFRP phase [11].

	Parameters	Symbol	Value
Material properties [11]	Density	$\rho (Kgm^{-3})$	1580
	Longitudinal Young’s modulus in–plane	$E_{11}\left(GPa\right)$	116
	Transverse Young’s modulus in-plane	$E_{22}\left(GPa\right)$	8.5
	Transverse Young’s modulus normal-to-plane	$E_{33}\left(GPa\right)$	8.5
	In-plane shear modulus	$G_{12}\left(GPa\right)$	3.26
	Transverse shear modulus in 1–3 plane	$G_{13}\left(GPa\right)$	3.26
	Transverse shear modulus in 2–3 plane	$G_{23}\left(GPa\right)$	2.16
	In plane Poisson’s ratio	$\nu_{12}$	0.20
	Transverse Poisson’s ratio in 1–3 plane	$\nu_{13}$	0.20
	Transverse Poisson’s ratio in 2–3 plane	$\nu_{23}$	0.28
	Longitudinal tensile strength in-plane	$X_{t}(MPa)$	1500
	Transverse tensile strength in-plane	$Y_{t}(MPa)$	27
	Transverse tensile strength normal-to-plane	$Z_{t}(MPa)$	27
	Longitudinal compression strength in-plane	$X_{c}(MPa)$	900
	Transverse compression strength in-plane	$Y_{c}(MPa)$	200
	Transverse compression strength normal-to-plane	$Z_{c}(MPa)$	200
	In-plane shear strength	$S_{12}(MPa)$	80
	Transverse shear strength in 1–3 plane	$S_{13}(MPa)$	80
	Transverse shear strength in 2–3 plane	$S_{23}(MPa$	$80^{*}$
	In-plane longitudinal thermal conductivity	$\lambda_{11}\left(Wm^{-1}K^{-1}\right)$	7
	Transverse thermal conductivity in-plane	$\lambda_{22}\left(Wm^{-1}K^{-1}\right)$	0.8
	Transverse thermal conductivity normal-to-plane	$\lambda_{33}\left(Wm^{-1}K^{-1}\right)$	0.8
	Specific heat	$C_{p}\left(J{Kg}^{-1}K^{-1}\right)$	1200
Cutting conditions [12]	Tool rake angle	$\gamma_{0}({}^{\circ})$	25
	Tool clearance angle	$\alpha_{0}({}^{\circ})$	10
	Tool-tip radius	$r_{c}(\mu m)$	5
	Specimen size	$\left({\mu m}^{3}\right)$	215×215×50
	Cutting speed	$V_{c}(m{min}^{-1})$	60
	Depth of cut	$a_{p}(\mu m)$	20
	Fibre orientation	θ(°)	0°,45°,90°,135°
	Friction coefficient	μ	0.68,0.84,0.63,0.96

* Jia et al. 2018 [4].

**Table 6:** Mechanical properties and Johnson–Cook parameters used for Ti6Al4V alloy [4].

	Constants	Symbol	Value
Titanium alloy properties	Density	$\rho \left(Kgm^{-3}\right)$	4430
	Young’s modulus,	$E\left(GPa\right)$	113
	Poisson ratio	ν	0.342
	Melting temperature,	$T_{m}\left({}^{\circ}C\right)$	1680
	Room temperature,	$T_{0}\left({}^{\circ}C\right)$	25
	Thermal conductivity,	$\lambda \left(Wm^{-1}{{}^{\circ}C}^{-1}\right)$	7
	Thermal expansion coefficient	$\alpha \left({{}^{\circ}C}^{-1}\right)$	$9.1\times 10^{-6}$
	Specific heat,	$C_{p}\left(J{Kg}^{-1}{{}^{\circ}C}^{-1}\right)$	546
Johnson–Cook constitutive model		A(MPa)	1098
		B(MPa)	1092
		C(MPa)	0.014
		n	0.93
		m	1.1
Johnson–Cook damage model		$d_{1}$	−0.09
		$d_{2}$	0.25
		$d_{3}$	−0.5
		$d_{4}$	0.014
		$d_{5}$	3.87

**Table 7:** Cutting parameters considered for the simulation of CFRP/Ti6Al4V hybrid composite [12].

Tool rake angle	$\gamma_{0}({}^{\circ})$	25
Tool clearance angle	$\alpha_{0}({}^{\circ})$	10
Tool-tip radius	$r_{c}(mm)$	$0.050^{*}$
Specimen size	$\left({mm}^{3}\right)$	3×3×0.8
Cutting speed	$V_{c}(mms^{-1})$	50**
Depth of cut	$a_{p}(mm)$	$0.20^{*}$
Fibre orientation	θ(°)	0°,45°,90°,135°
Friction coefficient at Tool/CFRP interface	$\mu_{Tool/CFRP}$	0.68,0.84,0.63,0.96
Friction coefficient at Tool/Ti interface	$\mu_{Tool/Ti}$	$0.5^{***}$

* Soldani et al. [50], ** Jia et al. [4], *** Isbilir et al. [51].

**Table 8:** Temperature distribution vs. fibre orientation.

	CFRP Plate	Ti Plate
θ=0°	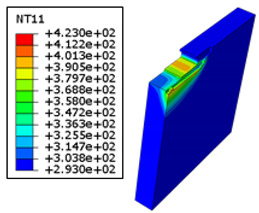	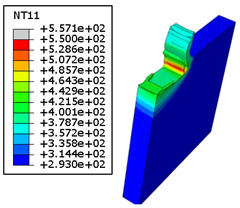
θ=45°	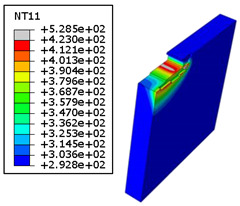	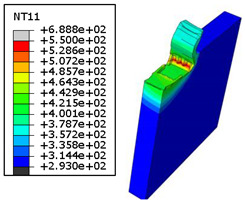
θ=90°	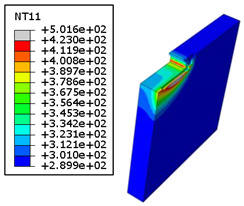	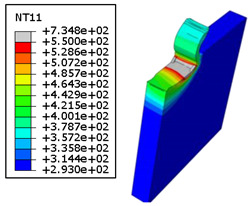
θ=135°	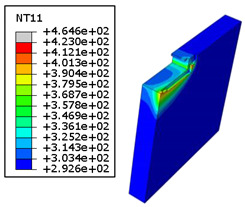	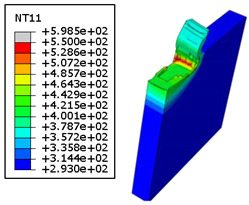

**Table 9:** Standard deviation SD calculated over $d_{sx}$ and $d_{sy}$.

SD	$d_{sx}(\mu m)$	$d_{sy}(\mu m)$
Ti	±2.93	±5.99
Interface	±6.96	±11.91
CFRP	±38.49	±89.09

## Data Availability

Not applicable.
